# Dominance of Fossil Fuels in Japan’s National Energy Mix and Implications for Environmental Sustainability

**DOI:** 10.3390/ijerph18147347

**Published:** 2021-07-09

**Authors:** Tomiwa Sunday Adebayo, Abraham Ayobamiji Awosusi, Seun Damola Oladipupo, Ephraim Bonah Agyekum, Arunkumar Jayakumar, Nallapaneni Manoj Kumar

**Affiliations:** 1 Department of Business Administration, Faculty of Economics and Administrative Science, Cyprus International University, 99040 Nicosia, Turkey; 2 Department of Economics, Faculty of Economics and Administrative Science, Near East University, 99138 Nicosia, Turkey; awosusiayobamiji@gmail.com; 3 Department of Earth Science, Faculty of Science, Olabisi Onabanjo University, Ago-Iwoye 110262, Ogun State, Nigeria; seunlad50@gmail.com; 4 Department of Nuclear and Renewable Energy, Ural Federal University Named after the First President of Russia Boris Yeltsin, 19 Mira Street, 620002 Ekaterinburg, Russia; agyekumephraim@yahoo.com; 5 Department of Automobile Engineering, SRM Institute of Science and Technology, Kattankulathur 603203, Tamil Nadu, India; arunkumj1@srmist.edu.in; 6 School of Energy and Environment, City University of Hong Kong, Kowloon, Hong Kong, China

**Keywords:** coal, CO_2_ emissions, economic growth, gas, Japan, natural, oil

## Abstract

Despite the drive for increased environmental protection and the achievement of the Sustainable Development Goals (SDGs), coal, oil, and natural gas use continues to dominate Japan’s energy mix. In light of this issue, this research assessed the position of natural gas, oil, and coal energy use in Japan’s environmental mitigation efforts from the perspective of sustainable development with respect to economic growth between 1965 and 2019. In this regard, the study employs Bayer and Hanck cointegration, fully modified Ordinary Least Square (FMOLS), and dynamic ordinary least square (DOLS) to investigate these interconnections. The empirical findings from this study revealed that the utilization of natural gas, oil, and coal energy reduces the sustainability of the environment with oil consumption having the most significant impact. Furthermore, the study validates the environmental Kuznets curve (EKC) hypothesis in Japan. The outcomes of the Gradual shift causality showed that CO_2_ emissions can predict economic growth, while oil, coal, and energy consumption can predict CO_2_ emissions in Japan. Given Japan’s ongoing energy crisis, this innovative analysis provides valuable policy insights to stakeholders and authorities in the nation’s energy sector.

## 1. Introduction

Climate change is a significant threat to mankind and global economic development. It also poses a significant challenge to stability, natural life, and growth. Climate change is largely caused by increasing greenhouse gas emissions (GHGs). Human practices, such as the use of non-renewable resources (NRE), contribute to GHG emissions, which in turn contribute to global warming [1]. The accumulation of CO_2_ emissions in the environment has increased, with far-reaching effects including flooding, violent storms, melting glaciers, droughts, and increasing sea levels [2]. CO_2_ emissions from the burning of fossil fuels lead to global warming [3]. Environmental pollution (primarily induced by the emissions of GHGs from economic activities) is causing increasing problems for world economies, prompting numerous policymakers to spend massive sums of monies to mitigate the risks [4]. This is due to the fact that both emerging and industrialized countries are increasingly being faced with the dual challenge of boosting economic output while simultaneously tackling environmental problems [5,6]. The primary target of policymakers in developing environmental policies differs around the world. It is unrealistic to expect all emerging nations to have the same ambition, which is to boost output at any cost, while ensuring environmental quality. Thus, politicians’ long-term solution for improving environmental sustainability is to develop long-term environmental policies [7].

This trade-off between balanced economic development and environmental destruction has been quantified in theoretical studies. Similarly, there are a number of scientific studies that have examined the determinants of environmental destruction and their causal interaction with economic development. For example, Kuznets [8] proposed the theory of economic growth and environmental decay, and later developed the inverted U-shaped curve. Furthermore, the study of Grossman and Krueger [9] identified positive relationship between economic growth and environmental degradation. Several reports on the trade-off between economic development and environmental destruction have been published, including, Umar et al. [10] for China, Adebayo et al. [11] for South Korea, Oluwajana et al. [12] for South Africa, Khan et al. [13] for Pakistan, Usman et al. [14] for the United States, Zhang et al. [15] for Malaysia, and Adebayo et al. [16] for Chile, among others.

**Figure 1:**
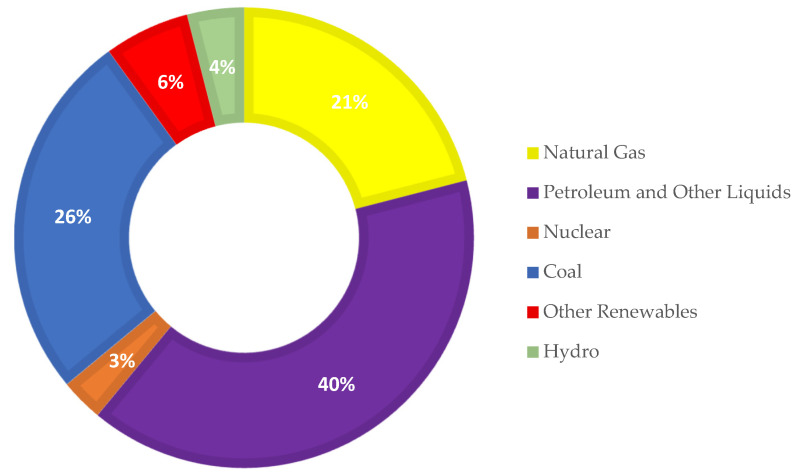
Japan Total Energy Consumption in 2019 Source: BP [17].

In 2019, Japan was the world’s fifth-largest oil consumer and fourth-largest crude oil importer. In the same year, Japan was also the globe’s biggest importer of liquefied natural gas (LNG) and the third-largest importer of coal, behind only China and India. As seen in Figure 1, fossil fuel constitutes about 87% of Japan’s energy mix, with petroleum contributing a high share (40 percent) of overall energy usage; nevertheless, natural gas and coal are becoming progressively crucial as sources of energy and have become the preferred options to compensate for the nuclear shortage [17]. In 2019, natural gas and coal accounted for 21% and 26% of gross primary use, respectively. The 2011 earthquake was responsible for this situation, because prior to this natural disaster, Japan was the globe’s third-largest consumer of nuclear power, behind only the United States and France, with nuclear power accounting for about 13% of total energy use in 2010. Its decarbonisation efforts were substantially hampered following the 2011 Fukushima nuclear accident, which forced it to abandon nuclear energy and increase its reliance on fossil fuels. For instance, by 2019, the nation’s nuclear energy share had reached 3%. This proportion is predicted to rise rapidly as more nuclear plants are restarted in the coming years [17].

In 2018, the majority of oil in Japan was consumed in the industrial (24%), non-energy consumption (16%), and transportation (38%) sectors. The power sector’s share has fallen from a peak of 19% in 2012 to 5% in 2018, when the sector started to substitute oil with alternative resources such as coal, natural gas, and nuclear energy. In 2019, naphtha, diesel, and gasoline accounted for the majority of the country’s oil product market. The impact of the current COVID-19 pandemic is projected to further weaken Japan’s appetite for petroleum products, especially diesel, gasoline, and jet fuel, with one of the most severe reductions in demand likely occurring during the first half of 2020 [18]. The COVID-19 crisis is now having a major impact on Japan’s economy. The effects of the pandemic are adding to an already diminishing pattern of GHG pollution, which fell by a yearly average of 2.5% between 2013 and 2018, and by 3.9% in 2018. With recent decisions to phase out obsolete coal-fired power plants and increase offshore wind power, the government’s present policies are expected to exceed its “very inadequate” 2030 Nationally Determined Contributions (NDC) goal, resulting in a performance that is still far from the Paris Agreement-compatible transition pathways [19]. Japan reiterated its commitment to reducing GHGs emissions by 26% below 2013 level by 2030 despite pressure from by the international community to increase its level.

Given the preceding impetus, it is critical to investigate the connection between CO_2_ emissions and coal use, natural gas, economic development, and energy use in Japan by providing answers to these questions: Do coal, oil, and natural gas contribute to environmental degradation in Japan? Is the EKC valid for Japan? What is the effect of economic expansion on environmental degradation in Japan? While a few researchers have explored similar scenarios, the current research can make a vital contribution to existing studies in a variety of ways. For example, the present research uses the key three energy sources in Japan, which are coal, nuclear power, and natural gas. As a result, the position of coal, oil, and natural gas use in Japan’s environmental performance is hypothesized. Furthermore, the environmental-income depletion interaction is reconsidered through the lens of the EKC hypothesis. To accomplish this, the present research speculates whether the EKC hypothesis probability in Japan is linked to the energy mix of the nation. As a result, aggregate primary energy usage is used to provide a novel insight that suggests that the N-shaped hypothesis is essentially viable for Japan’s case, particularly when energy consumption is primarily regarded. As a result of investigating the above mechanisms, the present research intends to substantially add to the established literature while also offering corrective steps to assist policymakers in establishing a more efficient potential connection between sustainable economic output and degradation of the environment.

The rest of this detailed analysis is structured as follows. Section 2 extensively discusses the literature viewpoints; Section 3 discusses the data, model, and methodology; Section 4 interprets the research outcomes; and Section 5 concludes with policy suggestions.

## 2. Literature Review

### 2.1. Theoretical Framework

The global economy has witnessed significant economic growth in the last four decades, including excessive energy use. Unfortunately, impressive economic growth and growing demand for energy have had environmental implications [20]. The study of Kraft and Kraft [21] was the first paper to establish the connection between energy use and economic growth. Ayobamiji and Kalmaz [22] claimed that it is difficult to achieve sustainable growth without increase in environmental degradation issues such as climate change and global warming. These types of issues have increased the awareness of environmentalists, economists, and policymakers, prompting them to explore the connection between GDP and environmental degradation. However, extensive studies have been done under the framework of EKC hypothesis, which was first confirmed in work done by Grossman & Krueger [23]. They confirmed that there is an inverted U-shaped connection between economic growth and environmental quality. The EKC hypothesis reveals that GDP contributes to environmental degradation and then reduces this impact when a threshold is reached in the economy [24]. However, there have been inconclusive outcomes regarding the validity of the EKC hypothesis.

### 2.2. Empirical Review

#### 2.2.1. Environmental Degradation and Economic Growth

Recent studies (such as [25,26,27,28,29] supported the validity of EKC hypothesis but was invalid in these studies (such as [15,30,31,32]. Furthermore, these studies appear to establish a different pattern of connection between economic growth and its environmental degradation, such as connection could be a U-shaped, N-shaped, and inverted-N shaped patterns which indicates that the environmental degradation could not be addressed automatically by economic growth. This indicates that the validity of EKC is partly due to reasons such as the proxy for environmental degradation, methodologies employed, the country or countries of investigation, and other related environmental indicators. For instance, the study of [33] found a positive association between CO_2_ emissions and GDP in 9 selected countries. The study of Ma et al. [25] in France and Germany established a one-way causal relationship between CO_2_ emissions and GDP.

Gyamfi et al. [34] employed the FMOLS and DOLS estimators to explore the relationship between CO_2_ emissions and GDP in 7 emerging economies and established that GDP increases environmental degradation. Adebayo & Akinsola [35] investigated the causal connection between CO_2_ emissions and GDP for the case of Thailand from 1971 to 2018. The investigators employed the Toda–Yamamoto causality techniques, conventional Granger, and wavelet coherence approaches to assess this association. Their empirical findings revealed one-way causality from economic growth to CO_2_ emissions. Furthermore, the wavelet coherence test outcome show a positive co-movement between GDP and CO_2_ emissions. The study of Khan et al. [13] asserted a positive connection between GDP and CO_2_ emissions between 1982 and 2018 in Pakistan. Bakhsh et al. [36] also inspected the association between CO_2_ emissions and GDP in Pakistan using the 3SLS model and discovered a negative relationship between GDP and CO_2_ emissions from 1980 to 2014. Hanif et al. [37] established a positive connection between CO_2_ emissions and GDP for 15 developing Asian nations. Mikayilov et al. [38] asserted a positive connection between CO_2_ emissions and GDP in Azerbaijan. Adebayo and Odugbesan [39] also established a positive connection between GDP and CO_2_ emissions for the case in South Africa between 1971 and 2016. The study of Awosusi et al. [40] revealed that CO_2_ emission is positively related to GDP from 1965 to 2016 for Brazil using ARDL and Gradual-shift causality approaches. The study of Adedoyin et al. [5] in BRICS nations between 1990 and 2014 revealed a positive connection between GDP and CO_2_ emissions. Using the Panel ARDL, [41] reported a positive connection between CO_2_ and GDP in 13 selected European nations over 1991–2014.

Adebayo et al. [42] established a one-way causal interconnection from GDP to CO_2_ emissions in Latin American nations. Khan et al. [33] reported a positive interaction between CO_2_ emissions and GDP using CS-ARDL, and the panel causality tests. The outcome reveals a one-way causal interconnection from GDP to CO_2_ emissions in seven selected OECD countries. Zhang et al. [15] discovered a unidirectional causal interconnection from CO_2_ emissions to GDP in Malaysia between 1960 and 2018. However, for 116 nations, [43] employed panel vector autoregression (PVAR) and generalized method of moment (GMM) to scrutinize the causal association between GDP and CO_2_ emissions utilising dats covering the period from 1990 to 2014 in 116 countries. Their finding revealed a bidirectional causal interaction between GDP and CO_2_ emissions. Ahmad et al. [44] established a unidirectional causal interaction from GDP to CO_2_ emissions in Croatia.

#### 2.2.2. Environmental Degradation and Energy Consumption

Adebayo and Akinsola [35] observed a two-way causal connection between CO_2_ emissions and energy use in Thailand. Muhammad et al. [45] concluded that the increase in energy use contributes to environmental pollution in 13 Muslim countries between 2002 and 2014. Furthermore, there is evidence of a one-way causal connection from energy use to CO_2_ emissions. Adebayo and Kalmaz [46] reported a one-way causal connection from CO_2_ emissions to energy use and also a positive relation between CO_2_ emissions and energy use in Egypt. Mahalik et al. [47] observed a unidirectional causal connection from energy use to CO_2_ emissions in selected BRICS countries between 1990 and 2015. Ahmed and Le [48] established the detrimental effect of energy use on environmental pollution in six selected Association of Southeast Asian Nations (ASEAN) and no causal interaction between CO_2_ emissions and energy use within the time span of 1996 to 2017. Chontanawat [49] reported in ASEAN countries and established a bidirectional causality between CO_2_ emissions and energy use. In Indonesia, [50] reported no causal interaction between CO_2_ emissions and energy use within the timespan 1985 to 2017 while [51] confirms a positive relation between CO_2_ emissions and energy use covering the period 1980 to 2016. Begum et al. [52] observed a positive interconnection between CO_2_ emissions and energy use, utilizing the ARDL, DOLS, and SLM U test between 1970 and 2009. The study of Magazzino [53] established a two-way causal relation between CO_2_ emissions and energy use.

#### 2.2.3. Environmental Degradation and Coal Consumption

The high carbon content in fossil fuels (such as coal) is heavily polluting, which then turns into CO_2_ emissions in the combustion phase [22]. The Fifth Report of the IPCC (Intergovernmental Panel on Climate Change) also affirmed that fossil fuel consumption is a major determinant of environmental degradation [54]. A recent study undertaken by [55] employed the ARDL covering the period between 1970 and 2015 for Indonesia disclosed a positive relationship between CO_2_ emissions and coal consumption. The study of [56] in South Africa on determinants of CO_2_ emissions revealed that the deterioration in the environment is caused by coal consumption. In addition, there is evidence of two-way causal relationship between coal consumed and CO_2_ emissions. Tiwari et al. [57] asserted that the connection between energy consumed and CO_2_ emissions was positive in India between 1966 and 2011. Shahbaz et al. [58] found that coal consumption induces CO_2_ emissions in South Africa between 1965 and 2008. Muhammad et al. [59] identified a positive interconnection between emissions of CO_2_ emissions and coal consumption in South Africa. Adebayo et al. [60] found a positive interaction between CO_2_ emissions and coal consumption in South Africa from 1980–2017.

Bloch et al. [61] found a two-way causal connection between coal consumption and CO_2_ emissions in China from 1965 to 2008. Pata [62] established in his study on Turkey a positive interconnection between CO_2_ emissions and coal consumed. Govindaraju and Tang [63] established a two-way causal connection between coal consumption and CO_2_ emission in India and China from 1965 to 2009. Lin et al. [64] uncovered a two-way causal relationship between coal consumption and CO_2_ emissions in India and China from 1969 to 2015. Al-mulali and Che Sab [65] discovered no causal relation between CO_2_ emissions and coal consumed for the top 10 coal-consuming nations within the timespan from 1992 to 2009. Shahbaz et al. [66] uncovered a one-way casual interconnection between coal consumption to CO_2_ emissions in India while a two-way casual interconnection between coal consumption and CO_2_ emissions in China during the period from 1971 and 2011.

#### 2.2.4. Environmental Degradation and Natural Gas

Dong et al. [67] explored the connection between natural gas and CO_2_ emission in China between 1965 and 2016 and the empirical analysis revealed a negative association between natural gas and CO_2_ emission and also a two-way causality connection between natural gas and CO_2_ emission. The study of [68] employed the panel data for 30 provinces in China from 2000 to 2015 to examine the connection between natural gas and CO_2_ emission. The empirical outcome revealed that in the eastern part of China, there is a U-shaped association in the western and central part of the country but for the eastern part of the country, there is an inverted U-shaped association. Dong et al. [67] found a negative interconnection between natural gas and CO_2_ emission in14 Asia-Pacific countries from 1970 to 2016 and a two-way causal interconnection between natural gas and CO_2_ emissions. Murshed et al. [69] reported that natural gas mitigates environmental pollution in Bangladesh between 1980 and 2015. Zambrano-Monserrate et al. [70] employed the ARDL and found a positive interconnection between natural gas and CO_2_ emission in Peru from 1980 to 2011. Azam et al. [71] confirmed one-way causation from natural gas consumption to CO_2_ emissions from 1990 to 2014.

#### 2.2.5. Environmental Degradation and Oil Consumption

The study of Al-Mulali [72] on the association between CO_2_ emission and Oil consumption revealed a bidirectional causality between CO_2_ emissions and oil consumption in the MENA economies. Alkhathlan and Javid [73] investigated the impact of oil consumption on Saudi Arabia’s environmental quality during the period from 1971 to 2013 and the empirical analysis affirms a positive association between CO_2_ emission and Oil consumption. This study of Alam and Paramati [74] scrutinized the causal interaction between CO_2_ emission and Oil consumption using VECM for the case of 18 selected developing countries between 1980 and 2012. The authors confirmed a two-way causal interaction between CO_2_ emission and oil consumption. Bildirici an Bakirtas [75] investigation in BRICTS examined the causal interconnection between CO_2_ emission and oil consumption and the outcome reveals a two-way causal interconnection underlying CO_2_ emission and oil consumption in Russia and Brazil; but for India, South Africa, China, and Turkey, the outcome reveals a one-way connection between CO_2_ emission and oil consumption.

However, this current study aims to explore the interaction between CO_2_ emissions and natural gas, and coal consumption, primary energy consumption and economic growth within the framework of EKC in Japan. This research will complement prior studies since it will fill existing gaps in energy or environmental literature by examining the impact of the energy mix on environmental quality within the framework of EKC in Japan.

## 3. Data, Model, and Methodology

### 3.1. Data

In this study, we utilised CO_2_ emissions (CO_2_) as the dependent variable, while the independent variables are oil consumption (OIL), primary energy consumption (PEC), natural gas (NGAC), and coal consumption (CC). All these variables were obtained from the British Petroleum database. Finally, gross domestic product (GDP) was obtained from the World Bank database was also used to achieve the main priority of this study. Furthermore, these variables were transmuted into their natural logarithm to reduce heteroscedascity. The metric unit and description of these variables were clearly stated in Table 1. This study covers the period from 1965 to 2019.

### 3.2. Model

The EKC hypothesis reveals the relationship between income (level of growth) and environmental degradation (emissions level), which is expressed in Equation (1) as

CO_2_ = f (GDP) (1)

where the environmental degradation is denoted as CO_2_ and income (level of growth) is denoted as GDP. Economic growth squared (GDP^2^) is expected to minimise environmental degradation. When the quality of the environment becomes an inferior good, the level of income will surpass the predetermined threshold income level (GDP^*^), indicating that the income is contributing to the quality of the environment. EKC function is defined as

CO_2_ = f (GDP, GDP^2^) (2)

CO_2_ = f (GDP, GDP^2^, PEC) (3)

Prior studies such as [49,50] concluded that economic growth and energy consumption are the major determinants of environmental degradation. Since the EKC hypothesis is a nonlinear connection between the CO_2_ emissions and economic growth, suggesting an inverted U-shape. Following the study of Ayobamiji and Kalmaz [22], it is expected that connection between the CO_2_ emissions and economic growth is positive. i.e., $\left(\beta_{1}=\frac{\partial {\mathrm{CO}}_{2}}{\partial \mathrm{GDP}}>0\right)$. However, the nonlinear relationship between CO_2_ emissions and economic growth is anticipated to be negative i.e., $\left(\beta_{2}=\frac{\partial {\mathrm{CO}}_{2}}{\partial {\mathrm{GDP}}^{2}}<0\right)$. For energy consumption, it is expected that primary energy consumption would exert a positive impact on CO_2_ emissions i.e., $\left(\beta_{3}=\frac{\partial {\mathrm{CO}}_{2}}{\partial \mathrm{PEC}}>0\right)$. However, this study also employed oil consumption, coal consumption, and natural gas consumption, which are fossil fuel sources. It is expected that these energy sources contribute to environmental degradation—i.e., $\left(\beta_{3}=\frac{\partial {\mathrm{CO}}_{2}}{\partial \mathrm{OIL}}>0\right)$; $\left(\beta_{4}=\frac{\partial {\mathrm{CO}}_{2}}{\partial \mathrm{CC}}>0\right)$ and $\left(\beta_{5}=\frac{\partial {\mathrm{CO}}_{2}}{\partial \mathrm{NGAS}}>0\right)$. This empirical framework for this study was based on [76] and [77] to examine the connection underlying oil, coal, and gas consumption and economic growth on CO_2_ emissions in Japan.

(4) $${\mathrm{CO}}_{2_{t}}=\beta_{0}+\beta_{1}{\mathrm{GDP}}_{t}+\beta_{2}{\mathrm{GDP}}^{2}{}_{t}+\beta_{3}{\mathrm{PEC}}_{t}+\varepsilon_{t}$$

The study remodified Equation (4) into Equation (5) by examining the importance of underlying energy sources used in Japan.

(5) $$CO_{2_{t}}=\beta_{0}+\beta_{1}GDP_{t}+\beta_{2}GDP^{2}{}_{t}+\beta_{3}OIL_{t}+\beta_{4}CC_{t}+\beta_{5}NGAS_{t}+\varepsilon_{t}$$

where GDP, GDP^2^, PEC, OIL, CC, NGAS, and CO_2_ denote economic growth, the square of economic growth, primary energy consumption, oil consumption, coal consumption, natural gas, and carbon emissions respectively. $\beta_{i=5}$ denotes the long-run elasticity of the considered variable and t indicates period.

### 3.3. Methodology

#### 3.3.1. Stationarity Test

It is essential to examine the series stationarity features, which is the first task in this current study. Therefore, this paper utilizes the Augmented Dickey Fuller (ADF) test proposed by Dickey and Fuller [78] and the Phillips-Perron (PP) test initiated by Phillips and Perron [79]. Several researchers have suggested that owing to the power difference of unit root tests regarding the size of the sample, it is vital to utilize more than one-unit root test to evaluate the integration order of the series. The key distinction between the ADF and PP tests is their sensitivity to serial correlation in error terms and heteroscedasticity. Equations (6) and (7) illustrate the ADF and PP tests, respectively

(6) $${\Delta Y}_{t}={\;\beta }_{1}Y_{t-1}+\overset{n}{\underset{i-1}{\sum }}b_{1}∆Y_{t-1}+\epsilon_{t}$$

(7) $${\Delta Y}_{t}={\;\beta }_{0\;}+{\;\beta }_{1}t+\beta_{2}Y_{t-1}+\epsilon_{t}$$

where the deterministic term vector is depicted by Y_t_, the error term, which is not serially correlated, is depicted by ε_t_.

In Equation (2), μ_t_ is I(0). In the PP test, heteroscedasticity in the error terms and serial correlation is ignored.

Also, since the empirical analysis covered a time timeframe that included the Asian financial crisis (1997), Japanese asset price bubble (1990–1991), global financial crisis (2008–2009) and Fukushima disaster (2011), the conventional unit root tests (ADF and PP) may yield misleading results. Thus, we employed the Zivot and Andrews (ZA) test initiated by Zivot and Andrews [80], which can simultaneously capture the stationarity features of the series and a structural break. The ZA test not only tests the unit root characteristics of each variable but considers one structural break. The ZA equation is depicted as follows

(8) $$\mathrm{Model}\;A:\;\Delta y\;=\;\sigma +{\;ûy}_{t-1}+\;\beta t\;+{\;\gamma \mathrm{DU}}_{t}+\sum_{j=i}^{t}d_{j}{\Delta y}_{t-j}+{\;\varepsilon }_{t}$$

(9) $$\mathrm{Model}\;B:\;\;\Delta y=\sigma +{\;ûy}_{t-1}+\;\beta t\;+Ɵ{\mathrm{DT}}_{t}+\sum_{j=i}^{t}d_{j}{\Delta y}_{t-j}+{\;\varepsilon }_{t}$$

(10) $$\mathrm{Model}\;C:\;\Delta y\;=\sigma +{\;ûy}_{t-1}+\;\beta t\;+Ɵ{\mathrm{DT}}_{t}+{\gamma \mathrm{DU}}_{t}+\sum_{j=i}^{t}d_{j}{\Delta y}_{t-j}+{\;\varepsilon }_{t}$$

where DU_t_ represents the dummy variable’s mean changes with likely break-period (TP) and the shift in the trend of the considered variable is represented as DT_t_. Model A, B, and C represents intercept model (K), trend model (T) and intercept and trend (K&T). Model C was generally considered during analysis. Formally,

(11) $${\mathrm{DU}}_{t}=\{\begin{array}{c} 1\ldots \ldots \ldots \ldots .\mathrm{ift}>\mathrm{TB}\\ 0\ldots \ldots ..\ldots \mathrm{ift}<\mathrm{TB} \end{array}\;\mathrm{and}\;{\mathrm{DU}}_{t}=\{\begin{array}{c} t-\mathrm{TB}\ldots ..\mathrm{ift}>\mathrm{TB}\\ 0\ldots \ldots ..\ldots \mathrm{ift}<\mathrm{TB} \end{array}$$

Since there is more than one structural change that occurs within the considered period. Therefore, the ZA unit could also produce an unreliable outcome so the need for a more advanced technique is required. For this cause, this study applied Lee & Strazicich unit-root test proposed by Lee & Strazicich [81] which is capable of capturing at least two structural shifts and can be defined by these subsequent equations. The data generation phase is reflected in Equation (12).

(12) $$Y_{t}={\;\theta }^{l}Z_{t}+{\;e}_{t,\;}e_{t}={\;\beta }_{\mathrm{et}-1}+{\;e}_{t,}$$

where exogenous coefficients is depicted as $Z_{t}$ with $\varepsilon_{t.}∼\mathrm{IID}\;N\left(0,\sigma^{2}\right)$. However, two models namely Fracture, and trend are undertaken in the presence of a structural fracture. In Equations (13) and (14), they are defined respectively. For the Fracture model, $Z_{t}$ is accepted as [1, t, D_1t_, D_2t_] but the occurrence of two changes are experienced when D_jt_ = 1 for $t\;\geq {\;T}_{\mathrm{Bj}}+1$, j = 1, 2, and 0.

(13) $$Y_{t}={\;\mu }_{0}+{\;\theta }_{1}\beta_{1t}+{\;\theta }_{2}\beta_{2t}+{\;y}_{t-1}+{\;v}_{1t,}$$

(14) $$Y_{t}={\;\mu }_{0}+{\;\gamma }_{t}+\theta_{1}D_{1t}+{\;\theta }_{2}D_{2t}+{\;v}_{2t,}$$

where $v_{1t,}$ and $v_{2t,}$ are the error term for Equations (13) and (14) and the trend of the variable is regarded as γ. Regression process of LS unit-roots are:

T-statistics of the LM unit-roots is depicted as Equation (16) with ∅=0 as the null hypothesis guarding the LS unit-roots.

(15) $$∆\mathrm{yt}\;={\;\theta }^{l}∆Z_{t}+\emptyset {\tilde{S}}_{t-j}+\overset{k}{\underset{i=1}{\sum }}{⋌}_{i}∆{\tilde{S}}_{t-j}+\varepsilon_{t}.$$

(16) $$\tilde{P}=T\bar{\emptyset }$$

In discovering the two endogenous breakpoints $\left(T_{\mathrm{Bj}}\right)$, Equations (17) and (18) were employed in the regression process.

(17) $${\mathrm{LM}}_{p}=\;\inf\tilde{p}\left(\tilde{\lambda }\right)$$

(18) $${\mathrm{LM}}_{p}=\;\inf\tilde{\tau }\left(\tilde{\lambda }\right)$$

where ${⋌}_{j}=\frac{{\mathrm{TB}}_{j}}{T}$, j = 1, 2, and T represents sample size. $\tilde{\tau }$ was used by the LM unit-roots to determine the estimated parameter.

#### 3.3.2. Cointegration Test

After the stationary properties of the considered variables have been established, the cointegration pattern can be examined. According to [76], many of the cointegration methods tend to produce unreliable outcome and conclusion which is contradicting. For example, in a comparative investigation done by [82] using [83,84]. From the analysis, a contradictory outcome was reported, in which the estimates of [83] confirmed the absence of cointegration but the presence of cointegration was established by Johansen [84]. Due to this difference in estimation, the Bayer and Hanck cointegration test was utilized by this study to determine the long run connection amongst the considered variable. The advantage of the Bayer and Hanck cointegration test is that it merges several cointegration tests such as [83,84,85,86]. cointegration tests but using the fisher formula. Therefore, Bayer and Hanck cointegration test is a combined test, which is defined as

(19) EG−JOH=−2[In(PEG)+In(PJOH)]

(20) EG−JOH−BO−BDM=−2[In(PEG)+In(PJOH)+In(PBO) In(PBDM)]

where PEG, PJOH, PBO, and PBDM are the level of significance [83], [84], [85], and [86], respectively.

#### 3.3.3. Long Run Coefficients Estimators

This study employed the FMOLS (Fully Modified Ordinary Least Square) estimator to explore the long-run coefficient interaction between CO_2_ emissions and its regressors. The FMOLS provides an optimal estimate during regression. It was initiated by [87], to address the autocorrection and endogeneity problem, thereby offering a robust estimate. The FMOLS is defined as

(21) $$Y_{i,t}={\;\sigma }_{i}+{\;\beta }_{1}X_{i,j}+{\;\varepsilon }_{i,\;t};\;\;\forall_{t}=1,\ldots \ldots .,T,\;\;\;i=1,\ldots \ldots .N$$

where $Y_{i,t}$ and $X_{i,j}$ are the dependent and independent variables respectively cointegrated with its slope ($\beta_{1}$). Whereas $\beta_{1}$ can either be homogeneous or not. Transforming the equation to be

(22) $$Y_{i,t}={\;\sigma }_{i}+{\;\beta }_{1}X_{i,j}+\overset{K_{i}}{\underset{k=-K_{i}}{\sum }}\gamma_{i,k}∆X_{i,t-k}+\varepsilon_{i,\;t};\;\;\forall_{t}=1,\ldots \ldots .,T,\;\;i=1,\ldots \ldots .N$$

$\xi_{i,\;t}=\left({\hat{\varepsilon }}_{i,\;t},∆X_{i,t-k}\right)$ and $\Omega_{i,\;t}=\lim_{T\to \infty }E\;\left[\frac{1}{T}\left(\sum_{i=1}^{T}\xi_{i,\;t}\right)\left(\sum_{i=1}^{T}\xi_{i,\;t}\right)\right]$ indicates the covariance in the long run and $\Omega_{i}=\Omega_{i}^{o}$ **+** $\Gamma_{i}+\overset{´}{\Gamma_{\iota }};\;\Omega_{i}^{o}$ depicts the covariance simultaneous nature and $\Gamma_{i}$ illustrates the weighted sum of autocovariance. Finally, the estimators of FMOLS as

(23) $${\overset{´}{\beta }}^{*}{}_{\mathrm{FMOLS}}=\frac{1}{N}\overset{N}{\underset{i=1}{\sum }}\left[{\left(\overset{T}{\underset{i=1}{\sum }}{\left(X_{i,j}-{\bar{X}}_{i}\right)}^{2}\right)}^{-1}\left(\overset{T}{\underset{i=1}{\sum }}\left(X_{i,j}-{\bar{X}}_{i}\right)Y^{*}{}_{i,j}-T_{{\overset{´}{\gamma }}_{i}}\right)\right]$$

(24) $$Y^{*}{}_{i,j}={\;Y}^{*}{}_{i,j}-{\bar{Y}}_{i}-\frac{{\hat{\Omega }}_{2,1,i}}{{\hat{\Omega }}_{2,2,i}}\;∆X_{i,t}{\;\mathrm{and}\;\hat{\gamma }}_{i}={\overset{´}{\Gamma }}_{2,1,i\;}+{\hat{\Omega }}^{0}{}_{2,1,j}-\frac{{\hat{\Omega }}_{2,1,i}}{{\hat{\Omega }}_{2,2,i}}\left({\overset{´}{\Gamma }}_{2,2,i\;}+{\hat{\Omega }}^{0}{}_{2,2,j}\right)$$

Furthermore, the DOLS (Dynamic Ordinary Least Squares) estimator was undertaken which serves as a substitute to FMOLS. It was initiated by Stock & Watson [88] and provide an efficient estimator that is asymptotic and also terminates from the regression process feedbacks. During the cointegration process, both the leads and lags and the error term are orthogonal in nature.

(25) $$Y_{t}={\;\sigma }_{i}+\;\beta {\overset{´}{X}}_{t}+{\overset{´}{D}}_{1t}D_{\overset{ˋ}{\gamma }1}\overset{r}{\underset{j=-q}{\sum }}∆X_{t\overset{ˋ}{+}j}\rho +v_{1,\;t};$$

It assumed that all the including the lead (r) and lags (q) to the differentiated regressors which remove correlations in the long run between $v_{1,t}$ and $v_{2,t}$.

#### 3.3.4. Gradual Shift Causality

The causality flow between CO_2_ emissions and its determinants was explored using the Fourier Toda–Yamamoto causality, developed by [89]. The advantage of this technique over the other or conventional causality test is its ability to account for a structural shift during the regression process, making it more accurate in terms of outcomes. However, this model was constructed on the VAR (p + d), which is illustrated in Equation (26).

(26) $$y_{t}=\;\alpha \left(t\right)+{\;\beta }_{1}y_{t-1}+\cdots +{\;\beta }_{p+\mathrm{dmax}}y_{t-\left(p+\mathrm{dmax}\right)}+\varepsilon_{t}$$

where the intercept of the VAR model is denoted α with the parameter of the coefficient depicted as β and parameters (CO_2_, EC, GLO, and URB) describes $y_{t}$. The definition of the Fourier Toda–Yamamoto causality is illustrated from Equation (26) to (25). Reference [89] employed the Fourier approximation, which is used to capture the structural shifts and it is defined in Equation (27) as follows.

(27) $$\sigma \left(t\right)={\;\sigma }_{0}+{\;\gamma }_{1}\sin\left(\frac{2\pi \mathrm{kt}}{T}\right)+{\;\gamma }_{2}\cos\left(\frac{2\pi \mathrm{kt}}{T}\right)$$

where the metric for change and size of the frequency is depicted as $\gamma_{2k}$ and $\gamma_{1k}$; the number of the frequency is s with the approximation frequency depicted as k. We calculate the Fourier Toda–Yamamoto causality in Equation (28), by substituting Equation (27) into Equation (26).

(28) $$y_{t}={\;\sigma }_{0}+{\;\gamma }_{1}\sin\left(\frac{2\pi \mathrm{kt}}{T}\right)+{\;\gamma }_{2}\cos\left(\frac{2\pi \mathrm{kt}}{T}\right)+\beta_{1}y_{t-1}+\cdots +{\;\beta }_{p+d}y_{t-\left(p+d\right)}+\varepsilon_{t}$$

This approach is guarded by the null hypothesis (H_0_: β_1_ = β_ѳ_ = 0) against alternate hypothesis (H_0_: β_1_ ≠ β_ѳ_ ≠ 0). Nazlioglu et al. (2016) employed the Wald statistic to test its hypothesis.

## 4. Results and Discussion

This section of the paper presents the outcomes based on the methodology employed as well as the discussion. The statistical properties of the considered variables are summarized in Table 2. The mean values of the variables are: CO_2_ emissions is (1049.088); GDP (34,658.28); PEC (17.41156); CC (3.493); OIL (222.203); and NGAS (55.984), while the median values are: CO_2_ emissions (1113.336); GDP (39,253.64); PEC (18.673); CC (3.250); OIL (230.884); and NGAS (55.311). However, the range of the dataset for the variables is CO_2_ emissions (446.904 to 1299.737); GDP (12,595.39 to 49,187.83), PEC (6.516 to 22.347), CC (1.965 to 5.096), OIL (87.936 to 270.506), and NGAS (1.826 to 124.752), while the standard deviation values for the variables used are: 210.376 for CO_2_ emissions; 10,924.96 for GDP; 4.154 for PEC; 1.059 for CC; 41.833 for OIL, and 40.305 for NGAS. Based on the normal distribution of the considered values, it is evident that all variables are normally distributed except OIL and PEC, given that the *p*-value of the Jarque-Bera is more than 0.01. After determining the statistical properties of the considered variables, the stationary properties can now be examined.

Table 3 and Table 4 summarize the stationarity characteristics of the considered variables. Table 3 reports the conventional unit root (ADF and PP) outcomes, which can be summarized as mixed order of integration at either I(1) or I(0), with CO_2_, GDP, PEC, CC, and NGAS integrated at I(1), while OIL is integrated at I(0). Based on our early argument about the inferiority of the results of conventional unit root tests with regard to their inability to incorporate structural breaks, thereby producing an unreliable outcome, this study applied the ZA unit root test, which is summarized in Table 4. The outcomes of the ZA unit root test indicated a mixed level of integration with GDP and OIL integrated at I(0), while CO_2_, PEC, CC, and NGAS are all integrated at I(1). Based on the aforementioned argument regarding the inferiority of the ZA unit root, this study applied the LS unit root test, the results of which are shown in Table 4. It shows that the variables have a mixed order of integration in which variables such as CO_2_, GDP, PEC, and NGAS are integrated as I(1), whereas CC and OIL are integrated at I(0). To confirm cointegration for Equation (5), this study applied the Bayer–Hanck cointegration test, which is reported in Table 5. The results show that null hypothesis can be rejected at a 5% level of significance, affirming the presence of cointegration amongst the considered variables. This shows there is a long-run interconnection amongst CO_2_, GDP, OIL PEC, CC, and NGAS.

After confirming the presence of cointegration amongst the considered variables, we proceed by examining the association between the variables of interest. Table 6 reveals the outcomes of FMOLS and DOLS estimators. According to Table 6, it is evident that OIL, CC, and NGAS are the underlying factors of CO_2_ emissions. The outcomes from the FMOLS and DOLS revealed that a 1% increase in the consumption of oil in Japan will cause CO_2_ emissions to increase by 0.655% and 0.650%, respectively, and this finding is consistent with the study of [73] in Saudi Arabia. Oil consumption now makes a greater contribution to environmental degradation compared to other fossil fuel sources. The possible explanation for this outcome is that oil was the most used fossil fuel in Japan within the considered period. This implies that a 1% upsurge in the consumption of coal in Japan will raise CO_2_ emissions by 0.384% and 0.392% as disclosed by both FMOLS and DOLS, respectively. This outcome aligns with the studies [55] in Indonesia, [62] in Turkey, [90] in India and [60] in South Africa. However, a 1% increase in natural gas consumption will cause CO_2_ emissions to increase by 0.068 and 0.064% as disclosed by both FMOLS and DOLS, respectively. This outcome is not surprising given the fact that Japan has a low production level and is also one of the major consumers of natural gas in the world; it depends on imports from other producing countries to satisfy virtually all of it demand for natural gas. In 2019, Japan was the world’s largest importer of liquefied natural gas (LNG). In April 2017, the deregulation of the natural gas retail sector began, which has attracted an increasing number of new market entrants to contend with regional incumbent natural gas providers. Roughly 13% of retail customers had switched vendors as of March 2020. However, the final stage of the deregulation of the natural gas sector that entails detaching the company’s transmission division from its production and distribution sectors will not go into effect until April 2022. This will make it difficult for the nuclear project to compete economically with natural gas for electricity production.This finding is in line with the studies of Xu & Lin [68] in the western and central provinces in China and [70] in Peru.

The estimated results reveal that for the association between GDP and CO_2_ emissions, the connection is positive, whereas a negative relationship is found between GDP^2^ and CO_2_ emissions. This confirms the validity of the EKC hypothesis in this model, indicating that the association between CO_2_ emissions vis-a-vis environmental degradation and income (growth) follows an inverted U-shaped trend. To be specific, from the estimators used, a 1% increase in the GDP contributes to the degradation of the environment by 3.919% and 3.666% as established by FMOLS and DOLS, respectively. However, increasing the square of GDP by 1% will reduce CO_2_ emissions by 0.438% (FMOLS) and 0.411% (DOLS). This finding is consistent with the studies of [25,26,27,28,29,91].

Furthermore, this study used Equation (4) as a robustness test for Equation (5). Equation (4) is intended to examine the association between CO_2_ emissions and GDP, while incorporating the GDP^2^ and primary energy consumption (aggregate of non-renewable and renewable energy). According to the results in Table 7, it is evident that the null hypothesis was rejected at a 5% significance level, indicating that there is cointegration amongst GDP, the square of GDP and CO_2_ emissions in Japan, establishing a long-run association between CO_2_, GDP, GDP^2^, and PEC.

However, the FMOLS and DOLS estimators reveal that the association between income (growth) and CO_2_ emissions follows an N-shape pattern, which validates the N-Shaped EKC hypothesis in Japan, as shown in Table 8. For FMOLS, a percentage change in GDP and square of GDP will CO_2_ emissions to change by −2.391% and 0.271%, while the DOLS outcomes reveal that emissions change by −3.517% (GDP) and 0.398% (GDP^2^), indicating that GDP improves the quality of the environment, while the square of GDP is harmful to the environment. The primary energy consumption (aggregate of renewable and conventional energy sources) is far more detrimental to the environment at higher proportions of 1.019 and 0.993 as indicated by FMOLS and DOLS, respectively. This shows that the energy mix of Japan contributes to environmental degradation. The possible reasons for these outcomes could be the state of the country’s renewable energy sources. As of 2006, nuclear energy contribution to power generation represented about 27.44%, which was the country’s largest source of electricity when compared to coal (24.60%), natural gas (22.12%) and oil (12.92%), and it was anticipated that the proportion of nuclear energy would increase by 13.56% and 22.56% in 2017 and 2030, respectively. However, this aspiration was dashed as a result of the earthquake and tsunami the struck the country in 2011, causing the facilities located in Fukushima to shut down causing the loss of six reactors capable of generating 10 GW of nuclear energy.

Since the long-run association between CO_2_ emissions and the regressors have been established, it is then necessary to examine the direction of the causality, which was achieved in this study by employing the Gradual Shift Causality test, as reported in Table 9. According to the estimations, there is a one-way causal connection from environmental pollution to GDP, indicating that CO_2_ emissions are a predicting variable of GDP. The causality of the major energy mix for Japan was also explored. For coal consumption, there is a unidirectional causality flowing from the consumption of coal to CO_2_ emissions in Japan. This finding indicates that coal consumption can provide a clear explanation of CO_2_ emissions in the future. This finding was corroborated by Shahbaz et al. [92] in India. For oil consumption, there is a bidirectional causal connection between oil and CO_2_ emissions, suggesting a feedback hypothesis. This clearly shows that oil consumption and CO_2_ emissions in Japan can predict each other. This is in line with the study of [74] on 18 selected developing countries and [72] on MENA economies. For natural gas, there is no causal interconnection between natural gas and CO_2_ emissions. Finally, there is a bidirectional causal connection between PEC and CO_2_ emissions, signifying a feedback hypothesis, which is consistent with the studies of Shan et al. [93] and Acheampong [43] on 116 countries.

## 5. Conclusions

Most countries’ economic policies are aimed at achieving long-term economic development. Nevertheless, economic growth may have an effect on global warming and climate change, two of the most pressing global challenges and concerns. Almost 10 years after the 2011 earthquake and the nuclear catastrophe in Fukushima, Japan has achieved considerable progress in achieving its aim of an efficient, robust, and sustainable energy system. It has diversified its energy mix and undertaken a comprehensive electricity and natural gas reform. Renewable sources have gradually expanded, nuclear power facilities have been reestablished and demands for energy efficiency have increased, causing GHGs emissions to decrease below 2009 levels. However, Japan’s energy mix remains one of the most carbon intensive among IEA members. Rapid progress is required on substantially reducing carbon in order to meet its newly declared goal of attaining carbon neutrality by 2050. Therefore, this study examines the influence of coal consumption, oil, natural gas and economic growth on CO_2_ emissions in Japan using a dataset covering the period from 1965 to 2019.

The present research applied the Bayer and Hanck cointegration test to assess the long-run association between CO_2_ emissions and the regressors. The outcomes of the cointegration test revealed a long-run association between CO_2_ emissions and coal consumption, oil, natural gas and economic growth. Furthermore, we applied both DOLS and FMOLS to capture the long-run association between CO_2_ emissions and coal consumption, oil, natural gas and economic growth. The outcomes of the FMOLS and DOLS showed that coal consumption, oil, economic growth, and natural gas trigger environmental degradation in Japan. Furthermore, the study validates the EKC hypothesis. For the total primary energy use framework, evidence of an N-shaped association was observed. Furthermore, we applied the Gradual Shift Causality test to ascertain the causal association between CO_2_ emissions and the regressors. The outcomes of the Gradual shift causality test revealed a one-way causal flow from GDP to CO_2_ and from CC to GDP, while a two-way causal interconnection was observed between CO_2_ and OIL, and PEC and CO_2_. Furthermore, no causal connection exists between CO_2_ and NGAS.

The outcomes of this research have aided us in reaching an agreement with the proponents of Japan’s energy intensity diversification. This can be accomplished by taking a more aggressive approach to renewable energy sources, which would help the nation maintain its economic momentum. From a policy perspective, the research revealed that Japan is heavily reliant on fossil fuels (non-renewables), as seen by the energy mix. The devastating consequences of growth environment could be mitigated by investing in and using renewable energy sources (e.g., wind, geothermal, solar, wind, etc.). The creation and execution of effective policies to control activity in Japan’s energy and industrial sectors will contribute to the nation’s long-term growth. If the government imposes CO_2_ emission restrictions on manufacturing companies and industries, this will help to reduce the country’s CO_2_ emissions. The potential of imposing penalties or hefty taxes on offenders of this legislation will deter environmental deterioration. Alternative (renewable) energy sources such as wind, hydropower, and oceanic energy sources should be introduced to promote energy conservation. The above-mentioned strategies will assist Japan in maintaining its good economic development and better environmental performance. Likewise, several scholars have recommended that technology such as Clean Coal Technology (CCTs) should be introduced into coal energy systems to improve efficiency and reduce GHGs emissions. As a result, bolstering research and development efforts will be critical in the introduction and implementation of new technologies for coal consumption in order to reduce CO_2_ emissions and achieve green growth and sustainable development.

Although the current research has yielded significant empirical findings in the case of Japan, one of the main limitations of this study is that CO_2_ emissions are viewed as the only proxy of environmental degradation. Additional research should be carried out by including other determinants of environmental degradation.

## Figures and Tables

**Table 1:** Definition of the variable.

Indicators	Description	Sourced
CO_2_	Carbon dioxide emission in million tonnes	British Petroleum database
OIL	Oil consumption in million tonnes	
PEC	Primary energy consumption in exojoules	
CC	Coal consumption in exojoules	
NGAC	Natural Gas consumption in million tonnes	
GDP	GDP per capita (constant 2010 US$)	World Bank Database

**Table 2:** Summary of the descriptive statistics.

	CO_2_	GDP	PEC	CC	NGAS	OIL
Mean	1049.088	34,658.28	17.411	3.493	55.983	222.203
Median	1113.336	39,253.64	18.673	3.250	55.311	230.884
Maximum	1299.737	49,187.83	22.347	5.096	124.752	270.506
Minimum	446.904	12,595.39	6.516	1.965	1.826	87.936
Std. Dev.	210.377	10,924.96	4.154	1.059	40.305	41.833
Skewness	−0.961	−0.463	−0.840	0.241	0.171	−1.318
Kurtosis	3.473	1.809	3.007188	1.572	1.770	4.663
Jarque-Bera	8.976	5.220	6.467	5.204	3.732	22.269
Probability	0.011	0.073	0.039	0.074	0.155	0.000
Observations	55	55	55	55	55	55

GDP: Gross domestic product; PEC: Primary energy consumption; CC: coal consumption; NGAS: Natural gas consumption.

**Table 3:** Conventional unit root outcome.

Variable	ADF		PP	
	Level	First Difference	Level	First Difference
CO_2_	−4.067	−6.030 *	−3.928	−6.052 *
GDP	−3.385	−5.398 *	−3.385	−5.204 *
PEC	−3.913	−5.465 *	−3.475	−5.500 *
OIL	−5.355 *	−4.225 *	−4.565 *	−4.117 **
CC	−2.478	−7.171 *	−2.590	−7.362 *
NGAS	−2.242	−6.117 *	−0.704	−6.072 *

* and ** depict significance level of 0.01 and 0.05. GDP: Gross domestic product; PEC: Primary energy consumption; CC: coal consumption; NGAS: Natural gas consumption; ADF: Augmented Dickey–Fuller (ADF) and PP: Phillips–Perron.

**Table 4:** Structural breaks unit roots outcome.

Variable	ZA Unit Root				LS Unit Root					
	I(0)		I(1)		I(0)			I(1)		
	T-stat	B1	T-stat	TI	T-stat	B1	B2	T-stat	BI	B2
CO_2_	−4.746	2009	−8.849 *	1983	−3.964	1991	2014	−8.465 *	1977	2007
GDP	−6.933 *	1988	−3.699	1993	−4.526	1975	1992	−6.925 **	1975	1990
PEC	−4.955	2001	−8.561 *	1983	−3.471	1976	1996	−6.781 *	1981	2007
OIL	−5.179 **	1994	−7.682 *	1983	−6.422 **	1978	2004	−4.206 *	1969	1973
CC	−4.315	1980	−7.679 *	1979	−6.367 **	1975	2008	−7.924 *	1975	1980
NGAS	−4.516	1978	−6.549 *	1981	−5.363	1982	2002	−4.530 *	1976	1983

* and ** depicts significance level of 0.01 and 0.05 correspondingly; TI: first structural break; TII: second structural break. GDP: Gross domestic product; PEC: Primary energy consumption; OIL: oil consumption; CC: coal consumption; NGAS: Natural gas consumption.

**Table 5:** Bayer–Hanck cointegration test.

Model	Fisher Statistics	Fisher Statistics	Cointegration Decision
CO_2_ = *f* (GDP, GDP^2^, CC, OIL, NGAS)	EG-JOH	EG-JOH-BAN-BOS	
	22.583 *	36.294 *	Yes
	CV	CV	
5%	10.576	20.143	

Note: * depicts significance level of 0.05. Engle-Granger, Johansen, Banerjee and Boswijk was denote EG, JOH, BAN, and BOS.

**Table 6:** FMOLS and DOLS estimation outcome.

	FMOLS			DOLS		
Variable	Coefficient	Std. Error	t-Statistic	Coefficient	Std. Error	t-Statistic
GDP	3.919 **	1.819	2.155	3.666 *	1.976	1.976
GDP^2^	−0.438 **	0.204	−2.152	−0.411 *	−1.966	−1.966
OIL	0.655 *	0.039	16.447	0.650 *	5.565	5.565
CC	0.384 *	0.026	15.044	0.392 *	4.111	14.111
NGAS	0.068 *	0.018	3.773	0.064 *	3.290	3.290
R^2^	0.998			0.999		
Adj R^2^	0.997			0.997		
S.E. of reg	0.003			0.003		

Note: * and ** denote 0.01, 0.05 and 0.1 respectively. GDP: Gross domestic product; PEC: Primary energy consumption; OIL: oil consumption; CC: coal consumption; NGAS: Natural gas consumption.

**Table 7:** Bayer–Hanck cointegration test.

Model	Fisher Statistics	Fisher Statistics	Cointegration Decision
CO_2_ = *f* (GDP, GDP^2^, PEC)	EG-JOH	EG-JOH-BAN-BOS	
	43.382 *	65.941 *	Yes
	CV	CV	
5%	10.576	20.143	

Note: * depicts significance level of 0.05. Engle-Granger, Johansen, Banerjee, and Boswijk was denote EG, JOH, BAN, and BOS; CV: critical value

**Table 8:** FMOLS and DOLS.

	FMOLS			DOLS		
Variable	Coefficient	Std. Error	t-Statistic	Coefficient	Std. Error	t-Statistic
GDP	−2.391 **	1.274	−1.877	−3.517 ^b^	1.534	−2.293
GDP^2^	0.271 **	0.145	1.869	0.398 ^b^	0.172	2.315
PEC	1.019 *	0.088	11.643	0.993 *	0.120	8.273
R^2^	0.980			0.987		
Adj R^2^	0.975			0.984		
S.E. of reg	0.010			0.010		

Note: *, ^b^ and ** denote 0.01, 0.05, and 0.1, respectively.

**Table 9:** Gradual shift causality test.

Causality Flow	Wald-Stat	No of Fourier	*p*-Value	Decision Rule
GDP → CO_2_	5.6629	3	0.5796	One-way causality
CO_2_ → GDP	29.0767 *	3	0.0001	
PEC → CO_2_	12.1543 ***	2	0.0955	Two-way causality
CO_2_ → PEC	14.9555 **	2	0.0365	
CC → CO_2_	12.4743 ***	1	0.0860	One-way causal link
CO_2_ → CC	5.7726	1	0.5665	
OIL → CO_2_	15.8493 **	1	0.0265	Two-way causality
CO_2_ → OIL	21.0585 *	1	0.0036	
NGAS → CO_2_	6.0681	2	0.5318	No causal link
CO_2_ → NGAS	8.8112	2	0.2664	

Note: *, ** and *** denotes 1%, 5% and 10% level of significance.

## Data Availability

Data is readily available at request.

## Abbreviations

ADF	Augmented Dickey–Fuller
BAN	Banerjee
BOS	Boswijk
CC	Coal consumption
CO_2_	Carbon emissions
DOLS	Dynamic Ordinary Least Squares
EG	Engle–Granger
EKC	Environmental Kuznets Curve
FMOLS	Fully Modified Ordinary Least Square
GDP	Gross domestic product
GDP^2^	Square of economic growth
GHGs	Greenhouse emissions
IPCC	Intergovernmental Panel on Climate Change
JOH	Johansen
K	Intercept model,
K&T	Intercept and trend
LS	Lee and Strazicich unit root test
NDC	Nationally Determined Contributions
NGAS	Natural gas
NRE	Non-renewable resources
OIL	Oil consumption
OLS	Ordinary Least Square
PEC	Primary energy consumption
PP	Phillips–Perron
SDGs	Sustainable Development Goals
T	Trend model
VAR	Vector autoregression
ZA	Zivot Andrews unit root test
